# CD34-Negative Malignant Renal Solitary Fibrous Tumor: Case Report and Diagnostic Insights

**DOI:** 10.7759/cureus.62366

**Published:** 2024-06-14

**Authors:** Madhurya Ramineni, Numbereye Numbere

**Affiliations:** 1 Pathology and Laboratory Medicine, University of Rochester Medical Center, Rochester, USA

**Keywords:** round cell sft, nephrectomy, staghorn vessels, bcor positive sft, spindle cell renal tumors, nab2-stat6, stat6, mesenchymal renal tumors, malignant solitary fibrous tumor, cd34 negative

## Abstract

Solitary fibrous tumors (SFTs) are rare fibroblastic neoplasms with diverse biological behaviors and widespread distribution. Primary renal SFTs are uncommon, and their malignant variants, especially those that are CD34 negative, are even rarer. This study presents a case of malignant renal SFT in a 57-year-old female, focusing on its immunomorphological features. On gross examination, the tumor's large size (11.5 cm) was remarkable. Microscopic analysis showed high cellularity, diffuse sheets of moderately pleomorphic ovoid cells, prominent staghorn vessels, tumor cell necrosis, and a high mitotic count. Immunohistochemistry revealed strong positivity for STAT6, vimentin, and Bcl-2 and, notably, negativity for CD34. The presence of the *NAB2::STAT6* gene fusion was confirmed through fluorescence in situ hybridization. This case emphasizes the need to consider SFT in the differential diagnosis of unusual renal tumors, even when CD34 is negative. The infrequency, morphological diversity, and resemblance to other tumors make diagnosing renal SFTs challenging. Accurate identification and classification as benign or malignant are crucial for proper clinical management and prognosis.

## Introduction

Solitary fibrous tumors (SFT) are rare mesenchymal neoplasms of putative fibroblastic origin. They are predominantly benign yet notable for local or distant recurrences in 10-30% of cases, even after many years of resection [1]. Historically, SFTs were believed to be primarily intra-thoracic or pleural-based lesions. Still, later evidence established a more widespread occurrence, with extra-thoracic tumors being, in fact, commoner than thoracic tumors [1].

Renal SFTs are particularly rare, with a reported prevalence of only 1.3% in a comprehensive nephrectomy series [2]. While most renal SFTs are benign, there have been a few malignant cases [3,4]. Often found incidentally on imaging, renal SFTs can be misidentified as the more typical renal cell carcinoma (RCC) due to non-distinctive radiological features [5]. The histopathological discovery of a mesenchymal renal neoplasm prompts a challenging differentiation from other rare spindle and mesenchymal lesions in the kidney area. Shared immunohistochemical features with other mesenchymal renal tumors can complicate diagnosis. CD34 negativity occurs in 5-10% of SFTs and is associated with high-grade characteristics such as round cell/epithelioid cytomorphology [6]. Correctly diagnosing renal SFTs and determining their benign or malignant nature is crucial for patient management. This report discusses a complex case of a CD34-negative malignant renal SFT in a 57-year-old female patient.

## Case presentation

**Figure 1:**
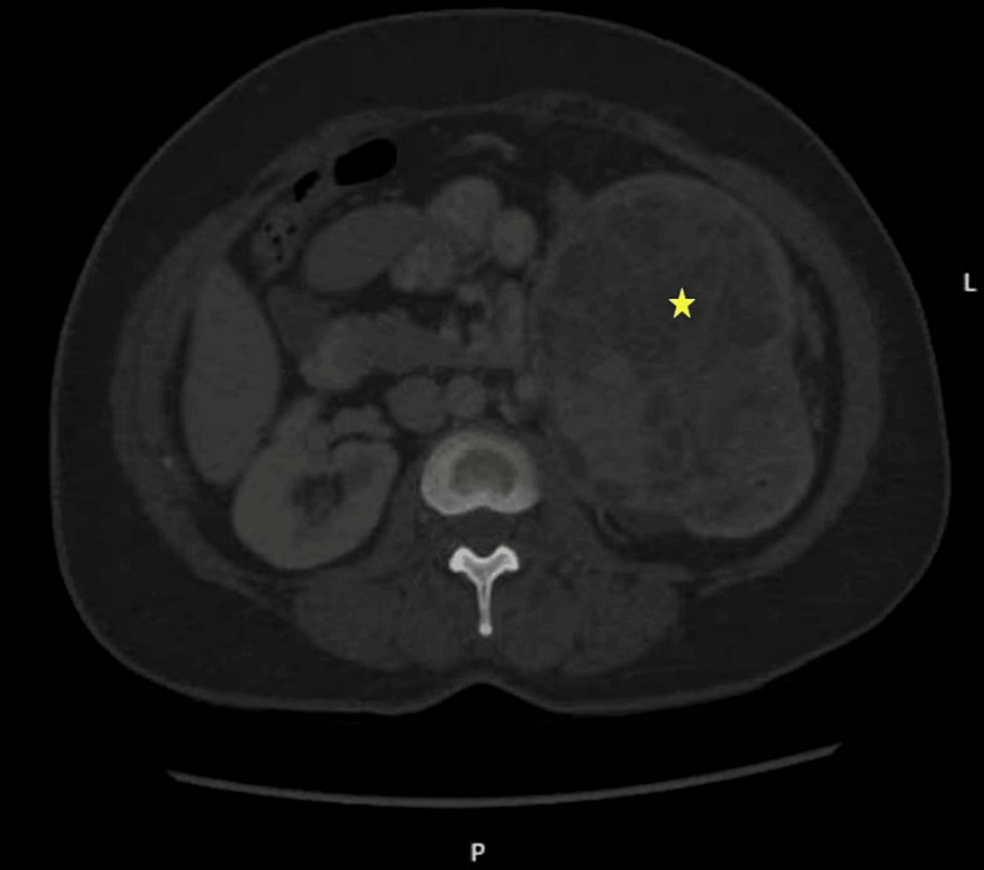
CT image Left kidney with a large intrarenal mass (yellow asterisk).

The patient, a 57-year-old woman, first presented to her primary care provider with a left intra-abdominal mass. Computed tomography (CT) imaging indicated that the mass originated from the left kidney (Figure 1).

**Figure 2:**
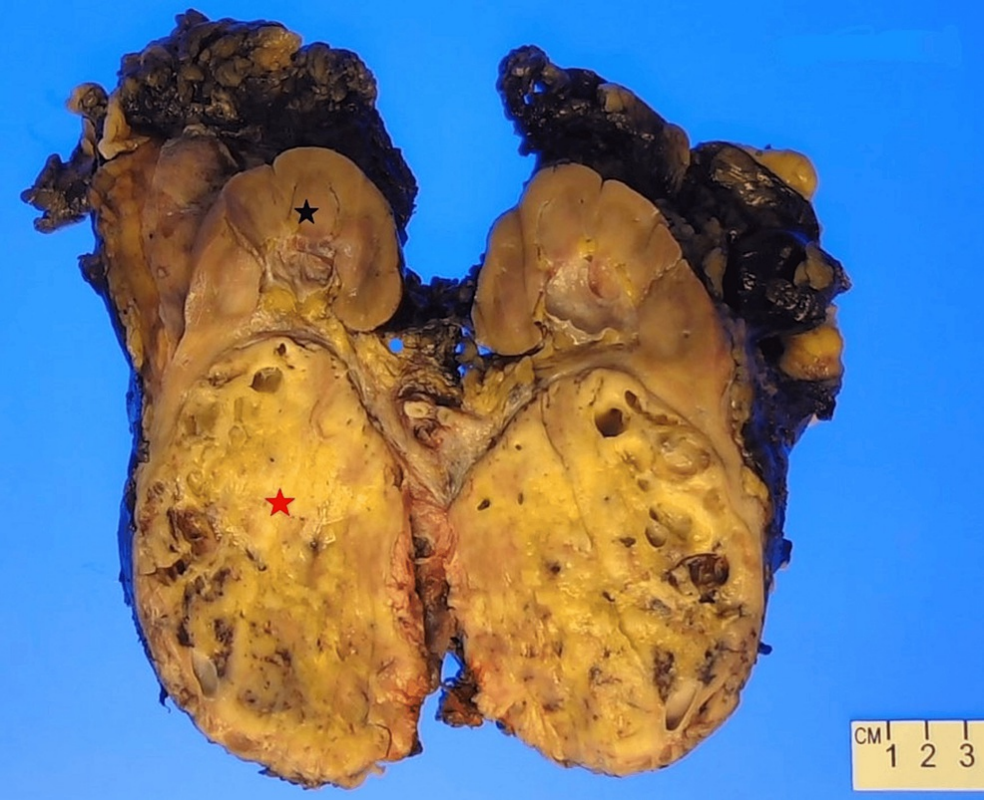
Gross pathology A large mass (red asterisk) replaces the renal parenchyma (black asterisk).

She underwent a robotic-assisted left radical nephrectomy due to a clinical suspicion of RCC. The gross pathology showed an 11.5 × 10 × 8 cm solid mass located at the lower pole of the kidney. The mass had a tan-pink to yellow coloration, with a consistency ranging from soft to rubbery. About 10% of the tumor demonstrated necrosis and areas of focal hemorrhage (Figure 2).

**Figure 3:**
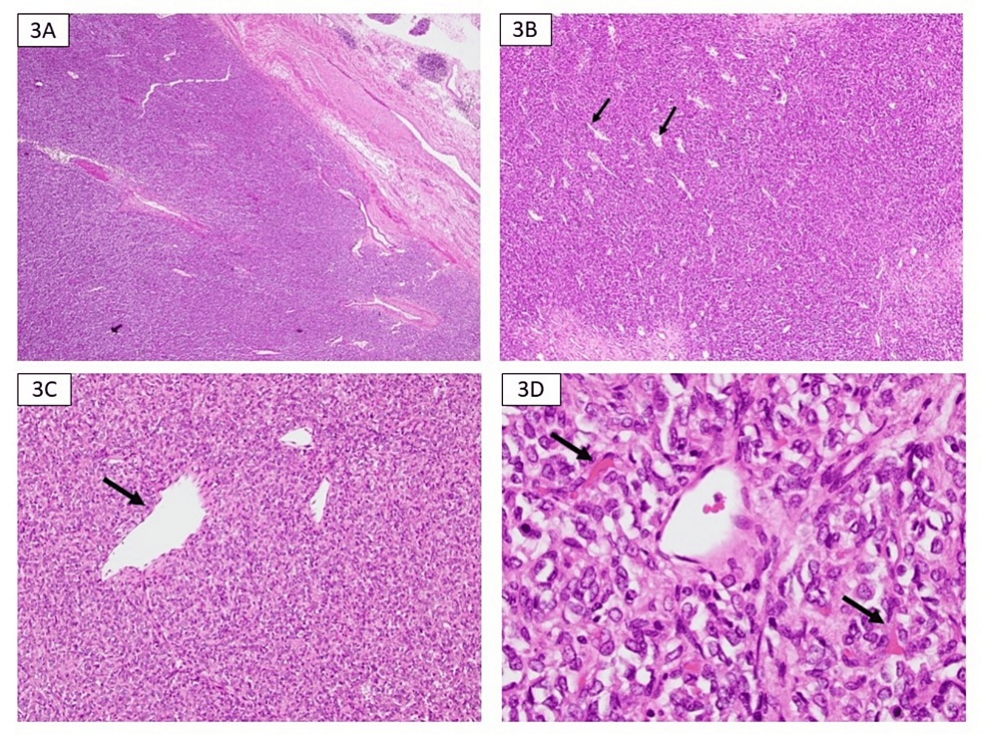
Microscopy, hematoxylin and eosin (H&E) stain 3A: 20× magnification demonstrating high cellularity; 3B: 40× magnification showing numerous interspersed staghorn-type vessels (black arrows); 3C: Prominent patulous blood vessels seen on 100× magnification (black arrows); 3D: 400× magnification demonstrating the ovoid nature of the cells set in a stroma with thin capillaries (black arrows).

**Figure 4:**
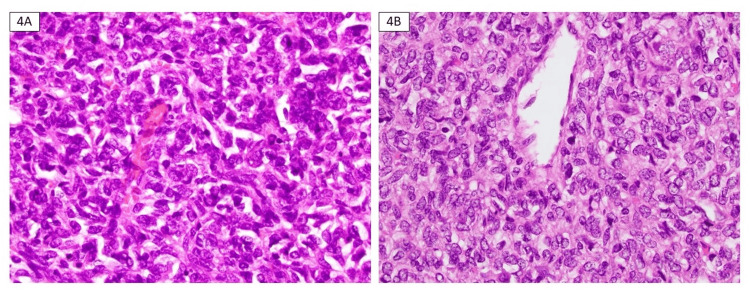
Microscopy, hematoxylin and eosin (H&E) stain 4A and 4B (400× magnification): Another high magnification image highlighting the sheets of ovoid cells with prominent blood vessels.

**Figure 5:**
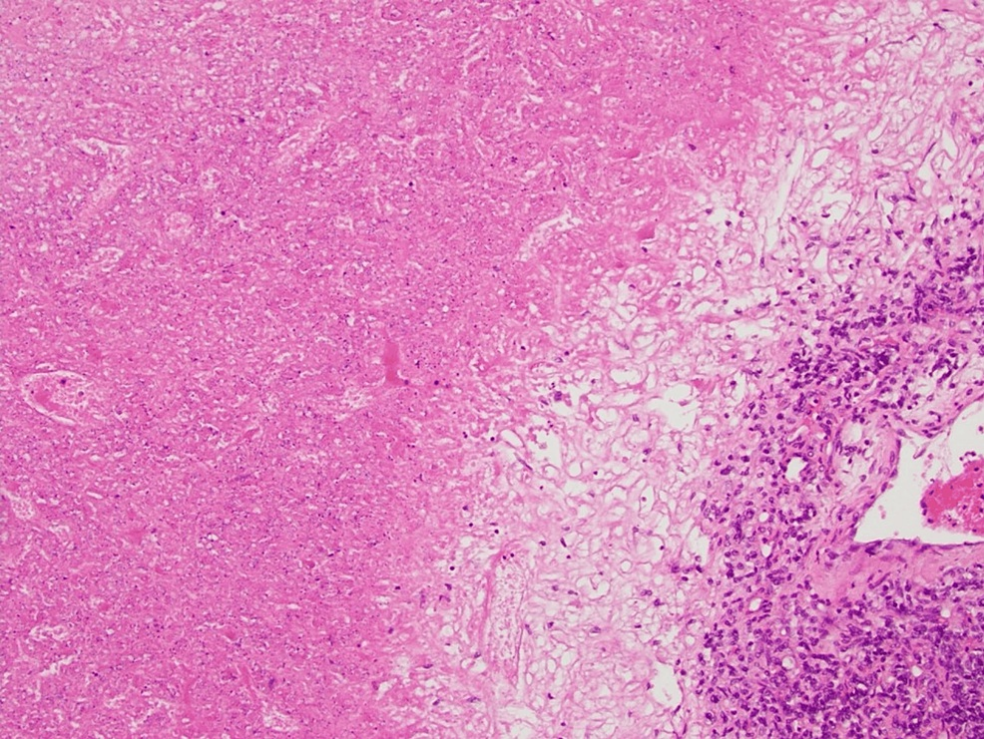
Area of tumor cell necrosis with viable tumor cells around an adjacent blood vessel

Microscopy revealed a circumscribed, highly cellular tumor consisting of ovoid cells arranged in sheets with moderately pleomorphic nuclei, moderate amphophilic cytoplasm, and conspicuous patulous blood vessels (staghorn vascular pattern). The neoplastic cells were embedded in a stroma featuring a dense network of fine blood vessels (Figures 3-4). The mitotic activity was noted at 7 per 10 high power fields (HPFs). Necrosis involving >10% of the tumor was identified (Figure 5). No lymphovascular invasion was present, and both lymph nodes and the adrenal gland were free of involvement. The neoplasm approached within less than 0.1 cm of the perinephric fat margin, the nearest margin to the tumor, without any evidence of renal vein or sinus fat invasion.

**Figure 6:**
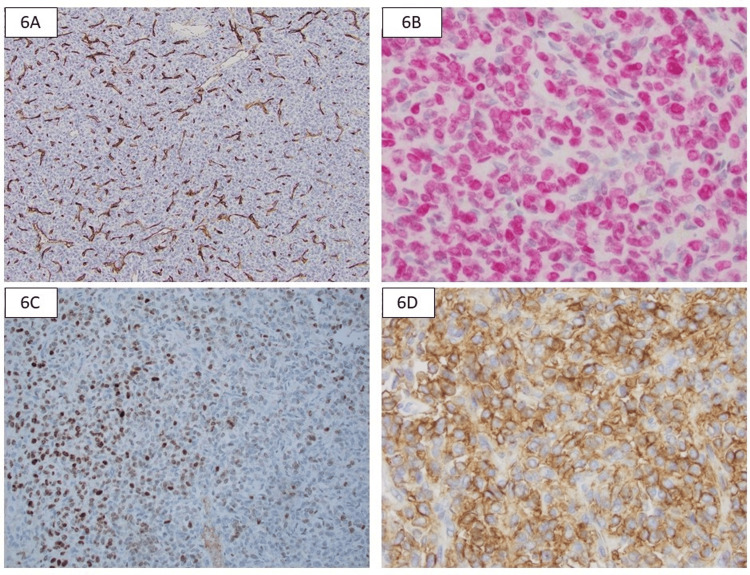
Immunohistochemistry 6A: CD34 stain highlights the interspersed vessels but is negative in the tumor cells; 6B: Strong and diffuse nuclear positivity for STAT6; 6C: Scattered cells show nuclear BCOR positivity; 6D: Membranous positivity for CD99 in the tumor cells.

The tumor cells were strongly positive for STAT6, vimentin, and Bcl-2. Moderate to weak scattered positivity was noted for both cyclin D1 and BCOR. CD99 showed nonspecific membranous/cytoplasmic positivity. The tumor cells were negative for cytokeratin AE1/AE3, SOX10, CD45, PAX8, CA IX, ERG, CD34, desmin, SMA, WT1, SATB2, and CD56 (Figure 6). Beta-catenin showed scattered membranous reactivity, which was interpreted as negative. *NAB2::STAT6* fusion was identified on fluorescence in situ hybridization (FISH) testing, confirming the diagnosis of SFT.

The morphology, immunohistochemical profile (positive staining for STAT6), and the characteristic *NAB2::STAT6* fusion were all consistent with SFT. Based on the histological features of ≥4 mitoses per 10 HPFs, tumor necrosis, and increased cellularity with atypia, the case was reported as malignant SFT. Based on the risk stratification criteria by Demicco et al., the tumor was given a score of six out of seven, placing it in the high-risk category for metastasis [7]. The SFT was localized at the time of diagnosis, so no chemoradiation was offered to the patient. She was placed on close surveillance with serial CT imaging.

## Discussion

Primary mesenchymal neoplasms of the kidney are rare and include diverse benign and malignant lesions. The prevalence of primary mesenchymal tumors in a large survey of nephrectomy specimens was 4.4%, with the majority being angiomyolipomas and renomedullary interstitial tumors [2]. Renal SFTs are rare, with most published literature in case reports and small case series [8,9]. Most renal SFTS are benign, with only a few malignant cases reported [3,4,10-14]. The first case of malignant renal SFT, which resulted in pulmonary metastasis, was reported in 2006 by Fine et al. [4].

Renal SFTs have been reported in patients ranging in age from four to 85 with no sex predilection [9]. The macroscopic appearance can be variable and is not characteristic of SFT. They can be well-circumscribed with lobulated contours, frequently bordered by a pseudocapsule, and possess a rubbery to firm tan-white to gray homogeneous cut surface [10]. They can also present with infiltrative borders, necrosis, hemorrhage, cystic changes, and prominent nodules [4].

The histopathologic and immunohistochemical features of renal SFTs are similar to their thoracic and extrathoracic counterparts [4,8]. On histology, most tumors show haphazardly arranged bland ovoid to spindled cells (“patternless pattern”) within a variably collagenized stroma with or without myxoid change. A notable feature is the presence of interspersed large, thin-walled, and ectatic (“staghorn”) vessels with perivascular hyalinization [8,10]. The typical immunohistochemical profile of benign renal SFTs is strong positivity for CD34 and STAT6, along with positive staining for Bcl-2 and vimentin in most cases [4,8].

SFTs may also display a wide range of morphologic diversity, thus leading to their designation by Machado et al. as the "great simulators of soft tissue tumors" [15]. In addition to the above-described classic spindled appearance, SFTs may display epithelioid and clear cell morphologies with nests of large epithelioid cells with abundant clear to eosinophilic cytoplasm [16]. Other cases may show focal or diffuse areas of hypercellularity that feature rounded lesional cells with large hyperchromatic nuclei and scant cytoplasm alongside sparse collagenous stroma, imparting a “small round cell” appearance to the tumor [15].

The histological features of ≥4 mitoses per 10 HPFs, necrosis, increased cellularity, and cytologic atypia define a malignant SFT [7,8]. However, the true biological potential and risk for metastasis in SFTs are difficult to predict despite these defining features of malignancy [7]. Therefore, the fifth edition of the WHO classification of bone and soft tissue tumors suggested using risk prediction algorithms over traditional terms like benign and malignant [17]. Although the patient did not show evidence of metastasis at the time of diagnosis, we reported the case as malignant SFT and commented on its high-risk nature per the risk stratification scheme by Demicco et al., which is exclusively validated for the risk prediction of distant metastasis but not local recurrence or survival rate [7].

Although CD34 reactivity is frequently seen in benign SFTs, its expression is often lost or weakly expressed with malignant transformation [3,4,6,9,10,17]. Our case of renal SFT showed increased cellularity and exhibited an ovoid cytomorphology rather than the classic spindly nature. The STAT6+, BCOR+, CD99+, and CD34 negative immunohistochemical profile seen in our case has been reported by Argani et al. and Lobo et al. to be associated with high-grade/dedifferentiated features [3,18]. Hypercellularity with round cell features is considered a high-grade feature and is observed in 48% of CD34-negative SFTs [6].

The pathologic differential diagnosis of renal SFT would vary depending on the histomorphologic appearance. In cases of renal SFT with the classic spindly tumor cells, the differentials to consider would encompass various benign and malignant spindle cell renal tumors such as sarcomatoid RCC, leiomyoma, leiomyosarcoma, dedifferentiated liposarcoma, and a range of other rare spindle cell sarcomas depending upon the degree of atypia. Conversely, a renal SFT with increased cellularity and a hint of epithelioid appearance, as in our case, prompts consideration of different sets of diagnostic possibilities. For the latter, the differential diagnoses include epithelial renal tumors like the various types of RCCs, epithelioid angiosarcoma, epithelioid sarcoma, epithelioid hemangioendothelioma, clear cell sarcoma of the kidney (CCSK), and various metastatic neoplasms depending upon the age, degree of atypia, and clinical background, among other factors [3,15,16]. BCOR immunohistochemistry, regarded as a sensitive and specific marker for CCSK, can also be expressed in renal SFTs, complicating the differential diagnosis. In the study by Argani et al., RNA-seq data indicated an upregulation of BCOR mRNA, suggesting a potential correlation between its expression and the risk of malignancy in SFT [3].

Management of SFT typically involves surgical resection, with complete resection being crucial due to the risk of recurrence, especially in high-risk or malignant cases. The role of adjuvant therapies is not well-established but may be considered in cases of incomplete resection or metastatic disease. Close follow-up and monitoring are recommended due to the potential for late recurrences [19].

## Conclusions

This case study focuses on a rare malignant SFT in the kidney of a 57-year-old woman. It underlines its atypical features, like increased cellularity, the non-classic ovoid/round cell morphology, loss of CD34, and BCOR positivity. The challenges in distinguishing renal SFTs from other renal neoplasms are demonstrated, highlighting the necessity of precise identification and risk assessment for optimal clinical management.
